# Cost-effectiveness analysis of the multi-strategy WHO emergency care toolkit in regional referral hospitals in Uganda

**DOI:** 10.1371/journal.pone.0279074

**Published:** 2022-12-14

**Authors:** Kalin Werner, Nicholas Risko, Joseph Kalanzi, Lee A. Wallis, Teri A. Reynolds

**Affiliations:** 1 Division of Emergency Medicine, University of Cape Town, Cape Town, South Africa; 2 Department of Emergency Medicine, Johns Hopkins University School of Medicine, Baltimore, MD, United States of America; 3 Makerere University School of Medicine, Kampala, Uganda; 4 Department for Clinical Services and Systems, Integrated Health Services, World Health Organization (WHO), Geneva, Switzerland; PLOS: Public Library of Science, UNITED KINGDOM

## Abstract

**Background:**

Low- and middle-income countries bear a disproportionate amount of the global burden of disease from emergency conditions. To improve the provision of emergency care in low-resource settings, a multifaceted World Health Organization (WHO) intervention introduced a toolkit including Basic Emergency Care training, resuscitation area guidelines, a trauma registry, a trauma checklist, and triage tool in two public hospital sites in Uganda. While introduction of the toolkit revealed a large reduction in the case fatality rate of patients, little is known about the cost-effectiveness and affordability. We analysed the cost-effectiveness of the toolkit and conducted a budget analysis to estimate the impact of scale up to all regional referral hospitals for the national level.

**Methods:**

A decision tree model was constructed to assess pre- and post-intervention groups from a societal perspective. Data regarding mortality were drawn from WHO quality improvement reports captured at two public hospitals in Uganda from 2016–2017. Cost data were drawn from project budgets and included direct costs of the implementation of the intervention, and direct costs of clinical care for patients with disability. Development costs were not included. Parameter uncertainty was assessed using both deterministic and probabilistic sensitivity analyses. Our model estimated the incremental cost-effectiveness of implementing the WHO emergency care toolkit measuring all costs and outcomes as disability-adjusted life-years (DALYs) over a lifetime, discounting both costs and outcomes at 3.5%.

**Results:**

Implementation of the WHO Toolkit averted 1,498 DALYs when compared to standard care over a one-year time horizon. The initial investment of $5,873 saved 34 lives (637 life years) and avoided $1,670,689 in downstream societal costs, resulted in a negative incremental cost-effectiveness ratio, dominating the comparator scenario of no intervention. This would increase to saving 884 lives and 25,236 DALYs annually with national scale up. If scaled to a national level the total intervention cost over period of five years would be $4,562,588 or a 0.09% increase of the total health budget for Uganda. The economic gains are estimated to be $29,880,949 USD, the equivalent of a 655% return on investment. The model was most sensitive to average annual cash income, discount rate and frequency survivor is a road-traffic incident survivor, but was robust for all other parameters.

**Conclusion:**

Improving emergency care using the WHO Toolkit produces a cost-savings in a low-resource setting such as Uganda. In alignment with the growing body of literature highlighting the value of systematizing emergency care, our findings suggest the toolkit could be an efficient approach to strengthening emergency care systems.

## Introduction

Emergency care (EC) refers to the systems and interventions that address acute illness and injury, such as trauma, infections, obstetric emergencies, and sudden complications of non-communicable diseases like diabetes, asthma, and cardiovascular disease. Health emergencies occur everywhere on a daily basis, whether or not there is an organised and well-resourced system present to address them. Over half of all deaths worldwide result from conditions amenable to emergency care, and the burden of these conditions is nearly 60% higher in low-income settings than middle or high-income settings [1, 2].

In response, the World Health Organization (WHO) established the Emergency, Trauma and Acute Care programme [3]. Based on identifying needs in low-resource settings (LRS), this unit developed and released a suite of tools to support the strengthening of emergency care systems. The simple package of interventions is designed to strengthen reliable, timely, efficient and effective management of patient care at facilities in LRS. The package includes a set of training and tools relevant to the provision of effective EC including: Basic Emergency Care (BEC) training for clinical staff, a trauma registry, standardised triage processes, emergency unit (EU) protocols and guidelines, trauma and medical checklists, and reorganization of the emergency unit (without addition of resources) to facilitate resuscitation of critically ill patients. All of the toolkit elements are freely available on the WHO website [4].

Like most LRS, Uganda faces a high burden of acute illness and injury, which increases the excess morbidity and mortality incurred by the lack of organised emergency care services. There are several local challenges in organising EC, including a lack of systemwide protocols, clinical documentation, equipment, and regulation [5]. A national assessment of facility-based acute care in Uganda identified inadequate EC in urban settings and no or minimal access to EC in rural settings [6]. Patients with emergency conditions in hospitals are commonly cared for by staff who have received no specialised training in EC and few sites have formal triage protocols to prioritise the delivery of care based on patient acuity, despite the established benefits of these features [7, 8].

The WHO Emergency Care Toolkit implementation was carried out with the Ministry of Health (MoH) at two Ugandan public hospitals from 2016–2017. Site selection was based on location to major thoroughfare, high volume of emergency visits, and support of hospital leadership. Both sites, Kawolo General Hospital (KGH) and Mubende Regional Referral Hospital (MRRH), are under management of the MoH, deliver services in a less formal manner than standard emergency units in high-income countries (HICs), and are staffed by non-rotating personnel who had not received specialised training in trauma and acute care prior to the intervention [9]. KGH is a 106 bed hospital located in the Central Region of Uganda and MRRH is a 175 bed hospital serving the districts of Mubende, Kiboga, Mityana and some of Mpigi District.

As part of these efforts, clinical process and outcomes including 48-hour case fatality rates, were collected for one year prior to (n = 2,241 patients) and one year after the intervention (n = 1,753) for all patients arriving with one of five sentinel conditions: road traffic injuries; paediatric pneumonia; paediatric diarrhoea; asthma, and post-partum haemorrhage. These conditions were selected due to the ease of rapidly assessing the impact of an EC intervention on outcomes. The mortality rate results of the analysis are reported in **Table 1**. Further details of how the WHO toolkit was introduced in Uganda and its impact are published elsewhere [9, 10].

**Table 1:** Model parameter range and probability distribution.

Parameter	Base case	Range	Probability distribution	Source
*Costs (USD)*				
Intervention costs	5873.80	4992.73–6754.86	gamma	Project budgets
Average annual cash income	1354.68	469.16–3,054.55	gamma	UBOSS [22]
Rehabilitation	27.03	22.97–31.08	gamma	Expert input
Prosthetics	672.5	400–945	gamma	Kenney et al [23]
Hospital care of sentinel conditions	15.19	12.91–17.47	gamma	Werner et al [24]
*Probability of 48hr case fatality prior to intervention*				
RTI	0.0314		beta	WHO QI Reports
PPH	0.0172		beta	WHO QI Reports
Asthma	0.0111		beta	WHO QI Reports
Paediatric pneumonia	0.0632		beta	WHO QI Reports
Paediatric diarrhoea	0.0267		beta	WHO QI Reports
*Odds ratios 48 hr survival post-intervention*				
RTI	0.265	0.1130–0.6216	beta	WHO QI Reports
PPH	0.000335	0–3.376E37	beta	WHO QI Reports
Asthma	0.00367	0–3.661E34	beta	WHO QI Reports
Paediatric pneumonia	0.423	0.2392–0.7481	beta	WHO QI Reports
Paediatric diarrhoea	0.5742	0.1495–2.2048	beta	WHO QI Reports
Survive without disability	0.962	0.818–0.992	beta	Chalya et al [25]
Survive with amputation	0.788	0.670–0.907	beta	Chalya et al [25]
Survive with neurological deficit	0.212	0.179–0.243	beta	Chalya et al [25]
Survive with rehab needs	0.115	0.098–0.133	beta	Chalya et al [25]
*DALY weights*				
Amputation	0.275	0.234–0.316	beta	Global Burden of Disease [26]
Neurological deficit	0.359	0.305–0.413	beta	Global Burden of Disease [26]
*Model assumptions*				
Discount rate (%)	3.5	0–5	beta	Egyptian Pharmacoeconomic guidelines [18]
Time horizon costs and outcomes	55	46.41–62.79	lognormal	Global Health Observatory [27]

CFR, case fatality rates; RTI, road traffic injury; PPH, post-partum haemorrhage; DALY, disability adjusted life years

While introduction of the toolkit revealed a promising reduction in the mortality rate for patients, little is known about the intervention’s cost-effectiveness and affordability [11]. These considerations are critical to decision makers and health system planners, who are operating under tight fiscal constraints. Research in this area has been highlighted as a critical gap and a recent systematic review demonstrated the paucity of published evidence around the cost-effectiveness of EC, particularly regarding analyses of multi-modal interventions that impact multiple diseases [12, 13]. Although cost-effectiveness analyses (CEA) should never be the sole criteria for decision making, this information can aid in making well-informed decisions regarding resource allocation, particularly in the setting of resource scarcity.

We present a CEA based on the results from Uganda and a budget impact analysis to estimate the affordability of scaling this to all regional referral hospitals at the national level.

## Methods

**Fig 1:**
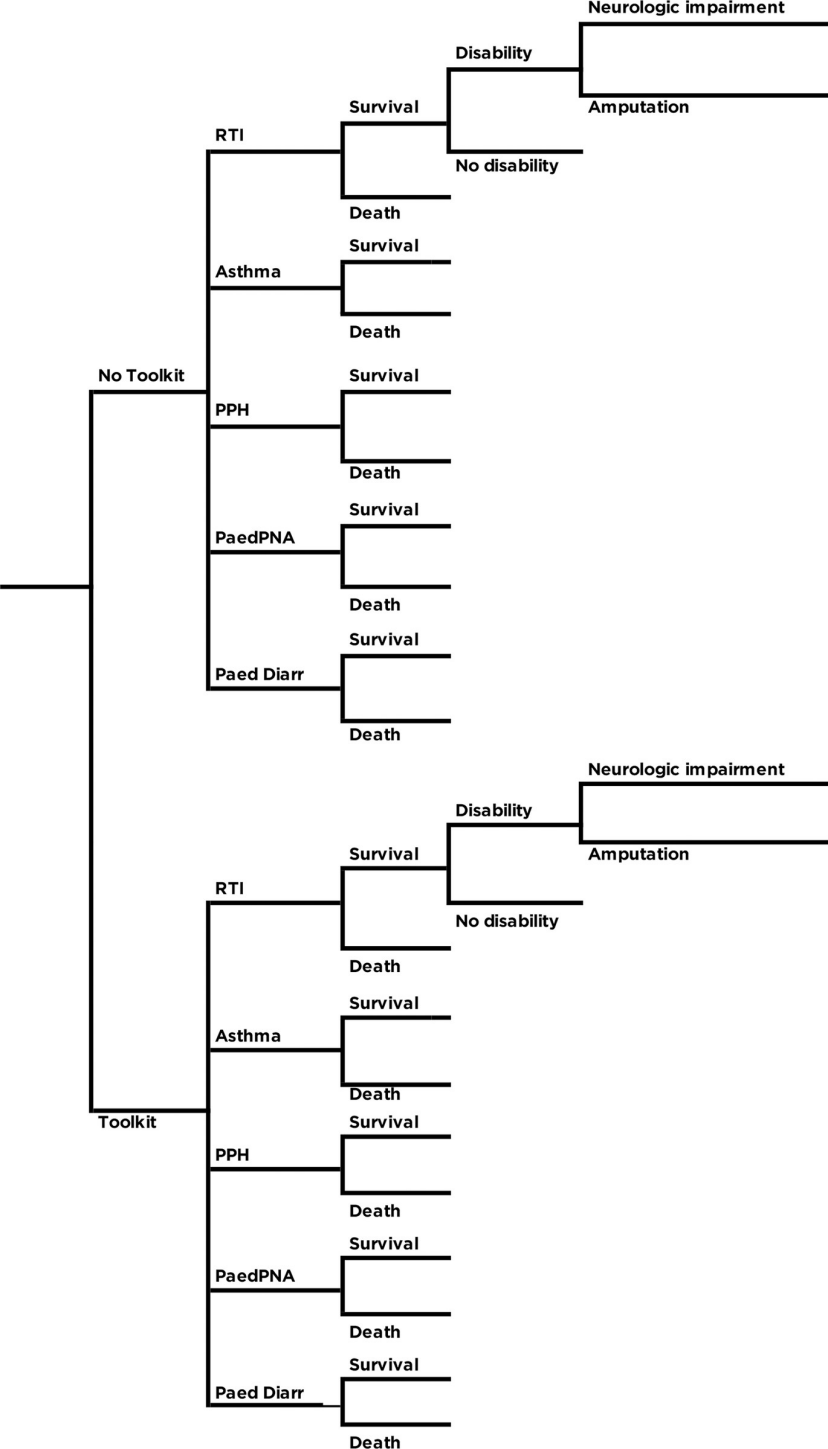
Decision tree model.

A decision tree model was constructed to compare the costs and health outcomes of implementing the package of interventions against the pre-intervention status quo from a societal perspective. In reporting our methods and results of the analysis we adhered to the Consolidated Health Economic Evaluation Reporting Standards statement (CHEERS) and ISPOR Principles of Good Practice for Budget Impact Analysis [14, 15]. The decision analytic model was developed using Microsoft Excel software (Microsoft Corp., Redmond, WA, USA) and is shown in **Fig 1** [16].

### Outcome measures and effectiveness

All probabilities related to clinical effectiveness were obtained from the WHO study [11]. The study population included patients presenting to either of the two selected hospital sites in Uganda for unscheduled care with one of the five sentinel conditions. Results were reported as 48-hour case fatality rates. Transition probabilities for post intervention parameters were derived by multiplying the probability of 48-hour case fatality in the pre-intervention group by the reported odds ratio.

We selected disability adjusted life years (DALYs) as our composite measure of effectiveness, given their wide acceptance as the primary measure for disease burden, particularly in low- and middle- income countries (LMICs) [17]. In addition, we reported incremental life-years saved (LYS) as a secondary unit of measurement to interpret effectiveness. These two measures allowed us to represent the circumstances of emergency conditions, which often impact the young during the peak of their economic productivity and enumerate the economic productivity losses resulting from premature death.

Both costs and health outcomes were discounted equally at a rate of 3.5% annually. This follows standard practice and published pharmacoeconomic guidelines from Egypt, the most relevant guidelines available for our setting [18].

In valuing health status, we used a human capital, rather than a preference elicitation approach. This follows prevailing practice for LMICs where health state valuation studies are uncommon [19]. Following this approach, value is appointed based on the economic activity of individuals and their ability to contribute to GDP [20]. Economic losses of production were estimated by the loss of income per year of life prematurely lost. The number of life years lost were enumerated by subtracting the median age of patients in the study population for each condition in the dataset (2 years for both paediatric conditions and 26 years for all other conditions) from the average life expectancy of the population at that age (68.6 years) [21]. All study parameters, including ranges and probability distribution used during monte carlo simulation, are outlined in Table 1.

### Resource use and costs

We used a micro-costing approach to estimate the resource use associated with introducing the toolkit. Indirect and direct costs were captured in 2017 Ugandan shillings (UGX) and converted to 2020 USD for reporting (1 US Dollar = 3,675 Ugandan shilling) [28].

A societal perspective was selected to allow for broad estimation of the intervention’s economic impact. Consistent with this, we tabulated all costs associated with the delivery of medical care, as well as costs borne by the public owing to the loss of income as a consequence of premature death and years of life lost. The majority of treatment costs were incurred within the first 48 hours of presentation at site, however for disabled survivors, costs and outcomes were evaluated over a lifetime horizon to capture all consequences, both financial and health related, assumed by society as a result of premature death or lifelong disability, including continued care costs for survivors with disability, and productivity losses due to premature death or disability. The time horizon of the intervention was limited to the available cohort captured during the study period of one year.

Costs of the intervention were derived by project budgets and through interviews conducted with research team staff [10]. Direct costs of implementation of the toolkit include: initial start-up training costs (training of trainer sessions), training in BEC for health care providers, and printing of posters and guidebooks for the triage tool triage, and trauma care checklists. Local trainers were first taught by international faculty involved in the development of the BEC course and country leads. In total across the two sites, four trainers were trained during training of trainer (ToT) sessions, ten health care providers were subsequently trained through roll out of the BEC course to each hospital site [10]. Costs for trainings included venue hire, skills equipment and supplies, printing, meals, per-diem reimbursement for attendees, and allowances of local trainers. Costs for international faculty were not included in our analysis since services were provided in kind. Furthermore, the WHO now supports various trainings annually provided by a network of BEC trained faculty free of charge. Five wall charts and 20 pocket manuals were printed with the triage tool. Average overall cost of training to the site was $1,683 for the ToT course and $3,515 for the BEC course (a total of $5,199 by site). All development costs of the toolkit materials, such as curriculum or checklist development, were provided in-kind by the WHO and are available free of cost for other interested countries who may seek to implement the toolkit intervention and therefore excluded from our analysis. Implementation of the toolkit required no additional space or equipment outside of what the hospital was already provided through existing national budget and procurement. The model assumed staff trained in BEC are already employed under the national staffing levels and therefore no additional salaries or human resources scale up are incorporated in to our analysis.

Hospital staff who had acted as local champions during the project were interviewed two years after the training to identify additional costs which may have occurred at the hospitals as a result of the intervention, such as continuing training or education for staff members. Although staff cited the use of a systematised approaches to onboarding new staff, no further costs were identified. Direct medical costs were enumerated by including both the costs of care in the treatment of the sentinel conditions, as well as expected medical costs of care required as a result of disability. The average cost of treatment for sentinel conditions was derived from a published micro-costing exercise of care emergency care interventions delivered in Uganda [24]. For example, survivors of road traffic incidents (RTI) with amputation included costs of immediate medical treatment, rehabilitation services, and prosthetic limbs. Additional care costs resulting from disability were valued by estimating the number of patients who survived with disability and multiplying that by an annual cost of care for each condition. The average cost was calculated and multiplied by the number of admitted patients during the data collection period. All costs used in the model are provided in Table 2.

**Table 2:** Summary of cost variables for the WHO toolkit intervention in Uganda.

Cost item	Price (2020 USD)
Start-up costs (including ToT)	$1,683
BEC training	$3,515
Triage tool	$172
Checklist tool	$503
**TOTAL**	**$5,873**

### Assumptions and threshold analysis

The thresholds used in this analysis make an implicit assumption of maximizing health across entire populations rather than seeking more equal health states across individuals [20]. We used additional guidance from Woods et al. regarding opportunity based cost-effectiveness thresholds for LMICs to consider our results at various levels of willingness to pay between $11 and $289 [29].

### Analytical methods

The study data did not stipulate the disability status of survivors, but we can assume that not all patients who survive are able to do so free of disability. Of the five conditions assessed, most are curable with appropriate treatment with the exception of RTIs which present a clear possibility of survival with disability, requiring further resources and care. Therefore, we assumed that patients surviving from all conditions, except RTIs, survive in full good health. To account for the impact of disability due to non-fatal injuries from RTIs we used published data on health outcomes for survivors of RTI to identify subgroups who would require further health costs after survival [25]. We then simulated the following outcomes proportionately based off of the literature; neurological disability (e.g. traumatic brain injury (TBI) or spinal cord injury) and musculoskeletal disability (e.g. loss of limb function/ amputation). Given the probability of disability was not directly measured by the study, we assumed that it was directly proportionate to survivorship, so if the intervention produced more survivors there would also be increased disability. Uncertainty around this assumption was explored in the sensitivity analysis. A micro-simulation was run to account for discounted costs and health outcomes. We assumed amputees require rehabilitation care services and renewed prosthetics every five years, and survivors with neurological impairment require life-long care. This care is frequently delivered by family members, rather than employed caretakers and therefore the cost of this care was quantified at the annual salary of one individual caretaker. We chose to use a conservative approach and include the loss of earning potential for family members responsible for care as a cost. To ensure we did not double count this measure, we avoid counting the loss of income as an additional cost to society. There is limited published data regarding return to work post-injury. However, we included additional loss of productivity for all survivors with disability, whom were assumed to not return to work after experiencing injury or disability.

### Scale-up and budget impact analysis

In LRS, interventions which prove cost-effective may still be prohibitively expensive [30]. To assess the affordability of the toolkit, we modelled a national scale up scenario to all regional referral hospitals (RRHs) allowing for projection of cost, impact and return on investment at a national level.

We presented the impact the rollout would have on both the total health budget and the budget for RRHs following ISPOR guidelines [15]. Using a payer perspective, we shifted our time horizon to 5 years with no discounting to assist with budget planning and forecasting [31]. Although primary data were not captured on the change in process functions, training may produce a net increased use of resources by health care workers as a result of gaining new skills, and knowledge through checklist reminders, or a net decrease due to mitigation of wastage [32, 33]. We took a conservative approach and modelled for a 15% increased utilization of resources arising from the intervention. This estimate was based on previous literature on the impacts of checklist implementation and provider training on care process measures in which trauma checklists result in an average 13% increase in frequency of selected list of process measures and 21% greater overall improvement in care quality measures [32, 33]. We assumed that treatment costs vary based on the number of patients seen and training costs fluctuate as a result of the number of sites, and when scaled fully the intervention would be implemented in all 19 RRHs.

A Return on Investment (ROI) analysis was performed by comparing the societal economic gains from having the toolkit at all RRHs with the current investment required to treat emergency conditions.

## Results

Implementation of the WHO Toolkit averted 1,498 DALYs when compared to standard care over a one-year time horizon. The initial investment of $5,873 saved 34 lives (637 life years) and avoided $1,670,689 in downstream societal costs. This resulted in a negative incremental cost-effectiveness ratio (indicating cost-savings), dominating the comparator scenario of no intervention. Results are presented in Table 3.

**Table 3:** Incremental cost-effectiveness results.

	Costs (USD)	DALYs	YLL	ICER $/DALY Averted	ICER $/LYS
No toolkit	24,607,487	20,919	1,178		
Toolkit	22,936,798	19,421	542		
Difference	-$1,670,689	1,498	637	-$1,115	-$2,624

USD, United States Dollar; DALY, disability adjusted life years; YLL, years of life lost; ICER, incremental cost effectiveness ratio; LYS, life-years saved

A national scale-up of this intervention to all RRHs over a five-year time horizon, would save 884 lives (10,780 life years) and 25,236 DALYs annually. Costs of the projected national scale-up are $4,562,588 USD, which would increase the Ugandan MoH budget by $570,862 USD over five years. This is equivalent to a 0.09% increase of the total health budget for Uganda based budgetary figures from 2018, or an increase of 1.64% of historic budgetary allotment specific to RRHs. The economic gains are estimated to be $29,880,949 USD, the equivalent of a 655% ROI. Details are provided in Tables 4 and 5.

**Table 4:** Budget impact analysis cost of national scale up to all RRHs.

Description	
*n* regional referral hospitals	19
Estimated annual EU patient population (for all RRHs)	38,000 or 2,000 per site
Deaths averted	884
LYS	10,780
DALY averted (non-discounted)	25,236
Costs of scale-up	$4,562,588 USD
Budget impact of scale-up	$570,862 USD
Health budget 2018/2019 [34]	2,363,652,000,000 UGX $643,941,532.62 USD
RRH budget 2018/2019	127,637,208,000 UGX $34,772,842.76 USD
Impact of scaling up on the total health budget	0.09% of total annual health budget
Impact of scaling up on the RRH budget	1.64% increase

EU, emergency unit; RRH, regional referral hospital; LYS, life-years saved; DALY, disability adjusted life years

**Table 5:** Budget impact of new treatment mix over five years.

Outcome	Year 1	Year 2	Year 3	Year 4	Year 5	*TOTAL*
*Cost outcomes new mix*						
Intervention costs	$111,602	$0	$0	$0	$0	*$111*,*602*
Treatment costs	$1,160,697	$ 829,153	$824,688	$820,344	$816,118	*$4*,*451*,*001*
						***$4*,*562*,*603***
*Cost outcomes old mix*						
Intervention costs	$0	$0	$0	$0	$0	*$0*
Treatment costs	$1,062,550	$738,722	$734,361	$730,118	$725,990	*$3*,*991*,*741*
						***$3*,*991*,*741***
*Budget Impact (BI)*						
Intervention BI	$111,602	-	-	-	-	
Treatment BI	$98,147	$90,431	$90,327	$90,226	$90,128	
**Total BI**	**$209,749**	**$90,431**	**$90327**	**$90,226**	**$90,128**	***$570*,*862***

### Addressing uncertainty

**Fig 2:**
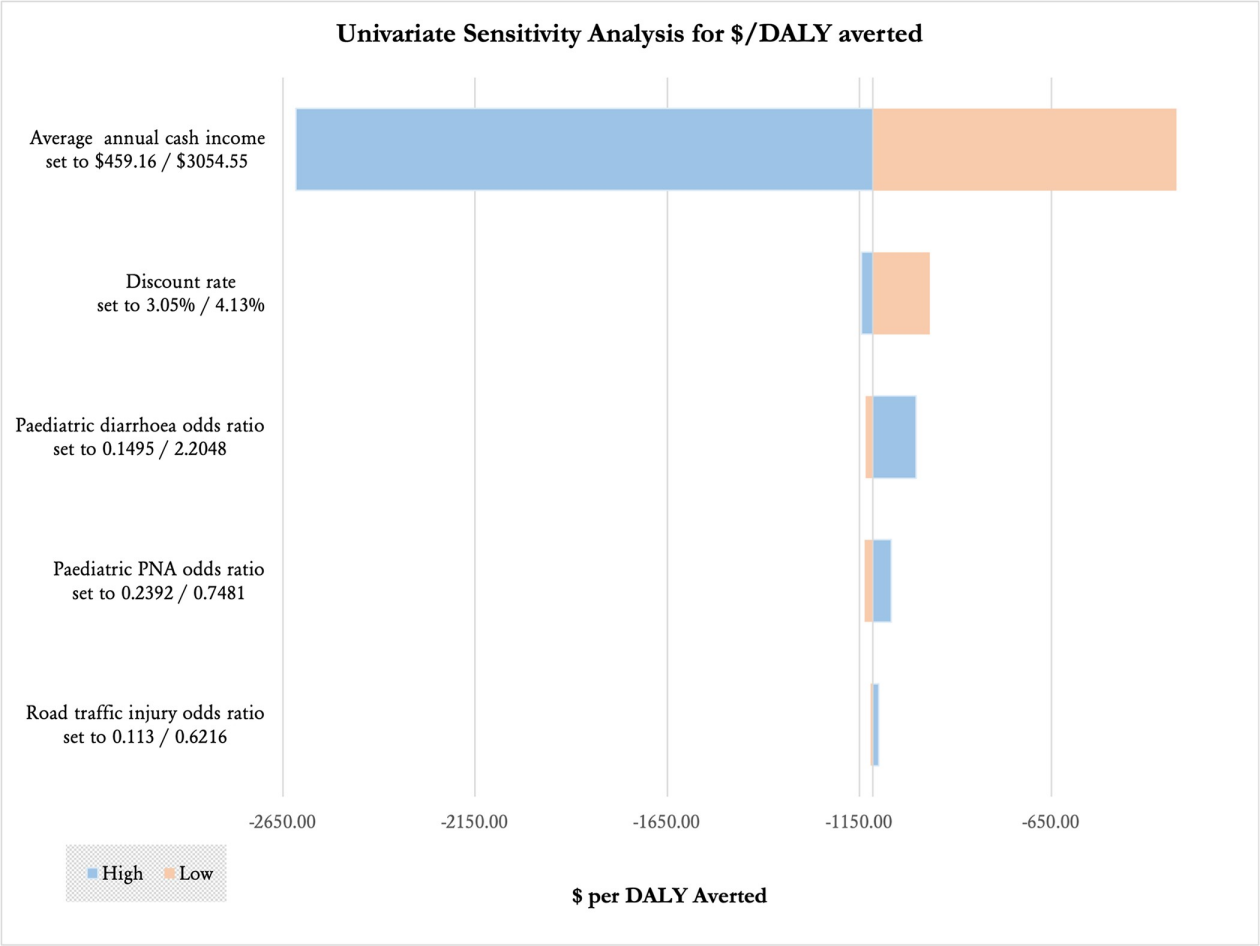
Results of a univariate sensitivity analysis of key variables on cost per DALY averted.

Parameter uncertainty was explored in two ways: first, with a univariate deterministic sensitivity analysis, and second with a multivariate probabilistic analysis and Monte Carlo simulation. Salaries were adapted to capture sub-regional variation from the lowest average monthly cash earnings (141,000 UGX per month or 1,692,000 UGX per annum in the Bukedi sub-region), to the highest earnings (938,000 UGX per month or 11,256,000 UGX per annum in the Kampala sub-region) [22]. Discounting rates were varied from 0% up to 5% to reflect current literature indicating that LMICs typically experience a higher economic growth rate and therefore require a larger discounting figure to accurately reflect the desire to make payments later on rather than now [35]. Average annual cash income, discount rate, and frequency survivor as an RTI survivor were the most vulnerable to changes. All other variables including costs associated with disability and frequency of surviving with disability were highly robust. Results of the deterministic sensitivity analyses are presented in **Fig 2.**

Additional sensitivity analyses were run on specific scenarios, including one in which the number of patients experiencing disability increased proportionally to the mortality benefit experienced by the intervention or approximately a 47% relative increase in morbidity. Under this scenario 197 fewer DALYs were averted, and $155,465 costs added. Under this scenario the intervention was more strongly dominant, contributing additional savings of $694 per DALY averted over the base case. Staff turnover rates could mean additional future training needs. We estimated a scenario in which the full costs of the toolkit would be bourne every five years. This change resulted in the intervention being only slightly less ($40) cost saving per DALY averted than the base case.

**Fig 3:**
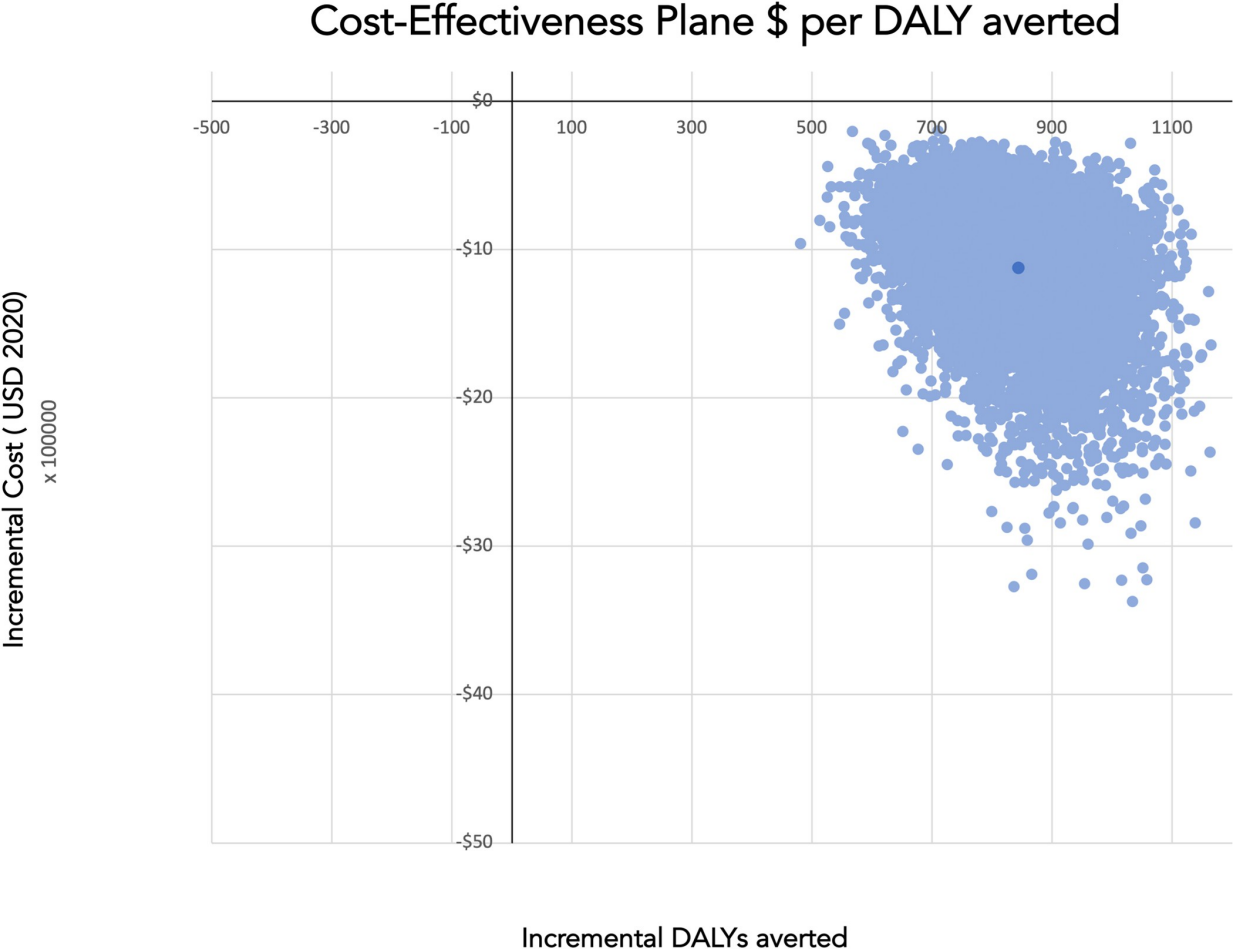
Cost-effectiveness plane results of 10,000 samples.

A probabilistic sensitivity analysis using Monte Carlo simulation was conducted to understand the interaction of multi-variable uncertainty. Distributions were assigned to each variable and simulations re-sampled 10,000 times varying all input parameters simultaneously. Uncertainty due to costs were assumed to take on gamma distribution which adequately represents the nature of cost as a continuous positive variable with a skewed distribution [36]. All probability parameters assumed a beta distribution. These results are presented in the cost-effectiveness plane in **Fig 3**.

The use of a cost-effectiveness acceptability curve to indicate the probability the intervention is cost-effective at various values of willingness to pay was not conducted given the extreme cost savings of the intervention. To assess the robustness of our assumption regarding increased service provision as a result of training, additional one-way sensitivity analysis was conducted for our budget impact analysis which showed our analysis was most sensitive to change in intervention volume and population per site.

## Discussion

This is one of the first CEAs to assess a multifaceted EC strengthening intervention introduced in a LRS [12]. Based upon the results of a WHO toolkit implementation, we compared the costs and effects of these improvements in RRHs in Uganda. Our results demonstrate with high certainty that the intervention is cost-saving, having a net positive health and economic impact. The intervention compares favourably to other common health programmes such as HIV treatment or rotavirus vaccinations ($890/DALY averted and $29/DALY averted respectively) which receive a substantial proportion of the current national health budget [37, 38]. Health care in Uganda faces extreme resource constraints with the country’s total expenditure on health per capita (Intl $, 2014) at $133 and health spending comprising only 6.4% of its GDP during the 2017/2018 reported year [21, 39]. Furthermore, our findings align with the growing body of literature indicating provider training and facility-based emergency care are cost-effective interventions in health [40–43].

This study sought to conduct a CEA utilizing the results of a completed study and further supported by local or contextually relevant data. As a result, our analysis has some important limitations. Firstly, effectiveness data were collected from two sites in Uganda with a sample size of at least 1500 patients in both pre-and post-intervention groups to detect at 30% mortality reduction. Therefore, caution is warranted when generalising the findings of this study to other country contexts. Furthermore, the scope of the data collected during the pilot study covered a small set of conditions meant to represent a varied group of mechanisms of illness and injury. Our results therefore give a close sense of the impact the toolkit may have, but cannot accurately estimate the complete value of the intervention if all emergency conditions and diseases were considered.

Small samples sizes were observed for certain conditions such as asthma and PPH and thus stratifying results by condition may not be accurate. However, the simulated population of our model mirrors the study population and is stratified by condition appropriately, thus the effect of the intervention on these two conditions likely has minimal impact on the results of our model.

The study outcome of 48-hour mortality was chosen by the WHO to isolate the impact of initial treatment and stabilization and avoid confounding from other conditions that may arise during prolonged hospitalization. However, it is possible that the intervention could continue to effect outcomes beyond this period, as available data suggests the quality of EU care can influence downstream costs and patient outcomes by decreasing length of stay or level of care, as well as reducing inpatient mortality [42, 44]. Regardless, the selection of 48-hour mortality as a primary outcome for the WHO study was outside the scope of this work and uncertainty around this was explored in our sensitivity analysis. It is possible that other external factors may be confounding the changes seen in mortality after the program was implemented. However, the clinical data analysis team adjusted all regressions for time and day of presentation. Condition-specific regressions were adjusted for key sociodemographic covariates (age and gender) and data was collected across a two year period allowing for inclusion of both wet and dry seasons to account for seasonal variation.

The implied cause of a change in health outcomes, which is not directly measured in this model, is the delivery of timely and appropriate care as a result of skills and knowledge gained through training and systematising approaches to triage. We suspect that training along with the use of checklists leads to an improved knowledge and comfort with principles of emergency care. Our base analysis was limited by a lack of empirical data on the difference between the frequency of treatment before and after the intervention. It is likely that supplies are being more appropriately used after the intervention, but the balance of decreased wastage and increased appropriate use was not measured. We addressed this by using current literature on the increased probability of treatment following training and checklist interventions to create an appropriate sensitivity analysis of a possible range which this change could account for. Without appropriately robust data we were not able to capture these changes in our analysis, although future research should seek to quantify the changes in practice of providers which may result from the intervention. Integrating key variables into regular QI data collection processes, such as procedure frequency or functional status at hospital departure for patient morbidity, could improve future assessments.

Furthermore, the true cost of disability was not measured in the study, nor is it well documented in the literature, particularly for LRS. Post-acute care in these settings is often provided by family members rather than through formal or institutional caregivers [45]. Despite the lack of data, we felt it was important to consider the economic burden of surviving with disability and include the losses that may be incurred in this scenario.

Finally, the available data did not allow for sub-analysis of the cost-effectiveness of different elements of the toolkit. It is possible that certain elements of the toolkit are not cost-effective and others are highly cost-effective, which on balance led to our results. However, improving skills and knowledge around resuscitation and triage, for example, will benefit all patients seeking emergency care regardless of their specific disease process. For this reason, measuring costs and mortality at the aggregate level has the advantage of catching economies of scope that may be achieved through emergency care interventions.

With regards to limitations inherent in the model, the sensitivity analysis demonstrates our model is highly sensitive to the variable of annual income. Using a human capital approach does not account for some of the other areas of life that may provide utility, and overlooks questions of equity and the intrinsic value of health. This choice also makes our model particularly sensitive to the input of average annual income. However, this approach also better captures the economic benefit of saving lives, and is consistent with many existing health technology assessment agency approaches [20]. It should be noted that many interventions in EC serve to benefit the young, who have the most potential for additional economic productivity throughout their lifetime. The large portion of paediatric patients in our study population is likely the reason our model is sensitive to this.

Finally, there are no data available on the sustained benefits of these organisational changes. Even though acute care may be primarily concerned with immediate actions, in taking a lifetime horizon approach it is essential to understand if further costs can be expected to maintain the impact witnessed from the studies. It is clear that strengthening this body of evidence could alter the outcomes of future CEAs. Despite the challenges, the knowledge produced through this research is a significant step forward in the field.

## Conclusions

This is a CEA that assesses a multifaceted EC strengthening intervention introduced in two Ugandan hospitals. The results indicate with a high level of certainty that the implementation of the WHO EC toolkit is cost-saving and leads to a net health and economic benefit. Budget impact analysis demonstrated that the small upfront investment to achieve this benefit is affordable in this setting, even when taken to the national scale. These findings support the adoption and scale-up of similar intervention packages in similar settings. Future research is needed to better understand changes in supply utilization after training and process interventions as well as the relative value of different elements of the toolkit.

## Abbreviations

BEC: Basic emergency care
EC: emergency care
ECSA: emergency care system assessment
LMIC: low- and middle- income countries
LRS: low-resource setting
MoH: Ministry of Health
HIC: high-income countries
QI: quality improvement
TBI: traumatic brain injury
ToT: Training of trainers
WHO: World Health Organization
